# RDAClone: Deciphering Tumor Heterozygosity through Single-Cell Genomics Data Analysis with Robust Deep Autoencoder

**DOI:** 10.3390/genes12121847

**Published:** 2021-11-23

**Authors:** Jie Xia, Lequn Wang, Guijun Zhang, Chunman Zuo, Luonan Chen

**Affiliations:** 1 College of Information Engineering, Zhejiang University of Technology, HangZhou 310023, China; xiajie@sibs.ac.cn; 2 Center for Excellence in Molecular Cell Science, State Key Laboratory of Cell Biology, Shanghai Institute of Biochemistry and Cell Biology, Chinese Academy of Sciences, Shanghai 200031, China; wanglequn2019@sibcb.ac.cn (L.W.); lnchen@sibcb.ac.cn (L.C.); 3 Key Laboratory of Systems Health Science of Zhejiang Province, Hangzhou Institute for Advanced Study, Chinese Academy of Sciences, University of Chinese Academy of Sciences, Hangzhou 310024, China; 4 Institute of Artificial Intelligence, Donghua University, Shanghai 201600, China; 5 Guangdong Institute of Intelligence Science and Technology, Hengqin, Zhuhai, Guangdong 519031, China

**Keywords:** single-cell genomics sequencing, deep learning, Louvain-Jaccard method, cell clustering, phylogenetic relationship

## Abstract

Rapid advances in single-cell genomics sequencing (SCGS) have allowed researchers to characterize tumor heterozygosity with unprecedented resolution and reveal the phylogenetic relationships between tumor cells or clones. However, high sequencing error rates of current SCGS data, i.e., false positives, false negatives, and missing bases, severely limit its application. Here, we present a deep learning framework, RDAClone, to recover genotype matrices from noisy data with an extended robust deep autoencoder, cluster cells into subclones by the Louvain-Jaccard method, and further infer evolutionary relationships between subclones by the minimum spanning tree. Studies on both simulated and real datasets demonstrate its robustness and superiority in data denoising, cell clustering, and evolutionary tree reconstruction, particularly for large datasets.

## 1. Introduction

Understanding the evolutionary mechanisms related to cancer progression and characterizing the intra-heterogeneity are promising routes for predicting and further controlling cancer progression, metastasis, and treatment responses [1,2,3,4,5]. Tumor tissue is composed of different subpopulations of cells with different genotypes, each called a subclone [6], and the evolutionary relationship of these subclones can be constructed based on the order of the mutated genes in these cells [4,7]. Many methods have been developed to deconvolute the bulk DNA sequencing data to identify subclones, and then construct the evolutionary tree [8,9,10,11,12,13]. However, bulk DNA sequencing data obtained from a mixture of millions of cells masks the properties of intra-tumor heterozygosity [14].

The rapid development of single-cell genomics sequencing (SCGS) offers an unprecedented opportunity to profile the evolutionary relationship between subclones in cancer tissue [15,16,17]. However, the application of current SCGS data has been severely limited by high-level experimental noise from single cell isolation, whole genome amplification, genome interrogation, allelic dropout events inducing false negative (FN) and false positive (FP) mutations, missing bases resulting from the insufficient sequencing coverage, and doublets from the mistaken selection of more than one cell [14,15]. Many studies have reported that the FP rate ranges from $3\times 10^{-5}$ to $7\times 10^{-5}$ and the FN rate ranges from 0.1 to 0.43. In addition, the reported missing rate (MR) is often more than 50% and up to 58% [18,19].

Recently, many computational methods have been developed to analyze SCGS data. For instance, SCITE [20] was designed to identify the evolution of the subclones of a tumor by modeling the sequencing errors based on a stochastic search algorithm. In addition, oncoNEM [21], a probabilistic model, was developed to infer intra-tumor evolutionary lineage trees from SCGS data. SCG [22], a hierarchical Bayesian model, has been proposed to simultaneously cluster cells into subclusters and infer corresponding genotypes, but it cannot be used to infer the evolutionary relations between these subclones. However, these three methods perform analysis tasks under the infinite site assumption, without considering recurrent mutations. SiFit [23] was proposed to infer tumor phylogenies from noisy SCGS data under a finite-sites assumption. BEAM [24] was developed to improve the quality in the SCGS data by using evolutionary information in the SCGS data in a molecular phylogenetic framework. However, these methods are difficult to scale to large datasets, especially the probabilistic models with exponential time complexity. To solve this problem, RobustClone [25] was designed to analyze SCGS data with the following three steps: recovering genotype matrices from noisy SCGS data by robust principal component analysis (RPCA) [26] or extended RPCA [27], clustering cells into subclones based on recovered genotype matrix by Louvain-Jaccard clustering [28,29], and finally, reconstructing a subclone evolutionary tree by finding the minimum spanning tree of these subclones. However, the biological data is inherently nonlinear and too complex to be represented by the linear model used by RobustClone, and the time complexity for RPCA is relatively high.

Deep neural networks have been widely applied to learn complex nonlinear features in many bioinformatics domains [30,31,32,33], including single-cell data analysis [34,35,36,37,38]. In particular, the deep autoencoder has been shown to be a computationally effective approach to extract the low-dimensional nonlinear features to accurately represent the data. Inspired by RPCA, the robust deep autoencoder (RDA) [39] was proposed to simultaneously represent the nonlinear features, which are robust to the noise and outliners [26], by minimizing the rank of low-rank matrices and the number of nonzero entries in the sparse matrix. Its optimization can be implemented by the alternating direction method of multipliers (ADMM) [40]. Recently, RDA has been widely applied in image denoising and anomaly detection [39,41,42]. These advantages of RDA show great potential to solve the difficulties in the current analysis of SCGS data.

Here, we present a computational framework RDAClone to analyze the noised SCGS data based on our extended RDA. Specifically, RDAClone can (a) denoise and impute the experimental SCGS data; (b) identify subclones from cancer cells; and (c) construct the subclone evolution trajectory. Our method can handle very large datasets even with high error/noise rates. To demonstrate its effectiveness, we applied the RDAClone method and other widely used methods to both simulated and real datasets, which demonstrated the superior performance of RDAClone compared to current state-of-the-art methods.

## 2. Methods

### 2.1. RDAClone Model

**Figure 1:**
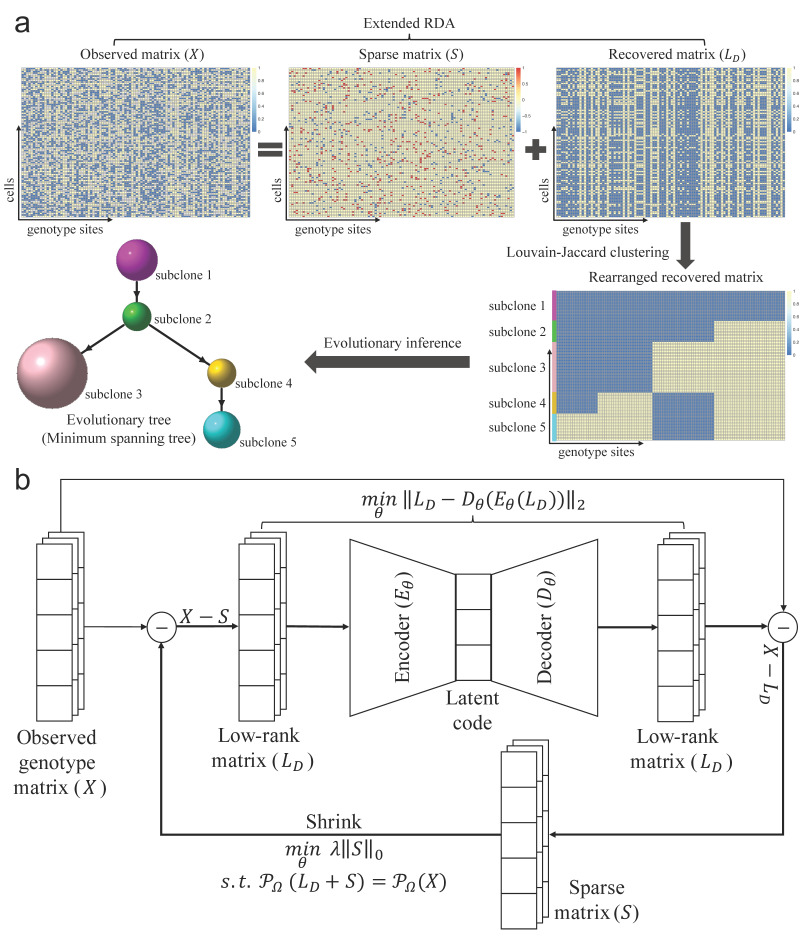
Overview of RDAClone framework and the computational graph for extended RDA model. (**a**) Given the SCGS data with row for cells and column for genotypes as input, RDAClone firstly decomposes the observed matrix (data) into a sparse matrix and a low rank recovered matrix by the extended RDA; clusters cells into subclones based on the recovered matrix by Louvain-Jaccard clustering method; and reconstructs subclone evolutionary tree by the minimum spanning tree of the subclone genotypes. (**b**) Recovering the genotype matrix by our extended RDA model as follows: first, initialize the low-rank matrix $L_{D}$ and sparse matrix S as zero matrices. Then, given an observed matrix X, the low-rank matrix $L_{D}$ calculated by X−S as the input of an encoder to extract the low-dimensional features, reconstructed back to the original dimensional space $L_{D}$ by a decoder, and the sparse matrix S calculated by $X-L_{D}$ is further optimized by a shrink operator to make S sparser. After the convergence of the computation, the recovered matrix $L_{D}$ generated by the extended RDA is kept for the subsequent analysis.

RDAClone was proposed to analyze SCGS data through the following steps: (1) decomposing the observed matrix into the sum of a low-rank matrix (i.e., a recovered genotype matrix) and a sparse matrix through our extended RDA; (2) clustering cells into subclones based on the recovered genotype matrix using the Louvain-Jaccard clustering method [28,29]; and (3) reconstructing a subclone evolutionary tree using the minimum spanning tree of the genotypes among these subclones [25]. The whole computational framework is shown in Figure 1a. Each method is described in detail below.

#### 2.1.1. RDA

Deep autoencoder learns the nonlinear low-dimensional features for the input data with an encoder and recovers the input data with a decoder, and the low-dimensional features can capture the information that accurately represents the input data. To process the experimental noisy data, RDA [41] was proposed to not only maintain a deep autoencoder’s ability to discover high-quality nonlinear features but also to eliminate the outliers by splitting the input data $\left(X\right)$ into a low-rank matrix $\left(L_{D}\right)$ and sparsity matrix $\left(S\right)$. This method has already been demonstrated to have powerful capability in image denoising and anomaly detection [39,41,42]. Hence, RDA is well adapted to process SCGS data due to its advantages over noisy data. The optimization objective of RDA can be summarized as two parts: minimizing the reconstruction loss of the of deep autoencoder, and the number of nonzero entries in sparse matrix S, which can be formulated as

(1) $$\begin{array}{c} \underset{\theta }{\min}\|L_{D}-D_{\theta }{\left(E_{\theta }\left(L_{D}\right)\right)\|}_{2}+\lambda \|{S\|}_{0}\\ s.t.\;L_{D}+S=X \end{array}$$

where $E_{\theta }$ and $D_{\theta }$ denote the encoder and decoder, respectively. S captures the noise information, θ is the learnable parameter in deep autoencoder, and λ is a hyperparameter to balance the two parts of the optimization objective, which can be fine-tuned manually. A larger λ makes the sparse matrix S sparser, while in contrast, a smaller λ will allow more noise to be isolated into S.

The above optimization is not computationally tractable. Following the idea in RPCA [27], the $\ell_{0}$ norm of S can be approximately replaced by the $\ell_{1}$ norm of S, and then the optimization problem takes the following form:

(2) $$\begin{array}{c} \underset{\theta }{\min}\|L_{D}-D_{\theta }{\left(E_{\theta }\left(L_{D}\right)\right)\|}_{2}+\lambda \|{S\|}_{1}\\ s.t.\;L_{D}+S=X \end{array}$$

#### 2.1.2. Extended RDA

To handle the amount of missing entries in the SCGS data, we proposed a novel algorithm named extended RDA (a nonlinear expansion of the extended RPCA [27]). We first define a projection as follows:

(3) $$P_{\Omega }\left(X\right)=\left\{\begin{array}{c} X_{ij},\;if\left(i,j\right)\in \Omega \\ 0,\;otherwise \end{array}\right.$$

The central idea of the extended RDA is to perform the decomposition only on the nonzero entries of the input SCGS data matrix. The missing entries are imputed to achieve a low rank in the recovered matrix, so that the optimization problem of the extended RDA can be formulated as follows:

(4) $$\begin{array}{c} \underset{\theta }{\min}\|L_{D}-D_{\theta }{\left(E_{\theta }\left(L_{D}\right)\right)\|}_{2}+\lambda \|{S\|}_{1}\\ s.t.P_{\Omega }\left(L_{D}+S\right)=P_{\Omega }\left(X\right) \end{array}$$

However, the above optimization problem is computationally infeasible. The constraint on nonzero entries is equivalent to penalize the nonzero entries in the sparse matrix S, and, thus, the extended RDA problem can be transformed into the following form:

(5) $$\begin{array}{c} \underset{\theta }{\min}\|L_{D}-D_{\theta }{\left(E_{\theta }\left(L_{D}\right)\right)\|}_{2}+\lambda \|P_{\Omega }{\left(S\right)\|}_{1}\\ s.t.L_{D}+S=X \end{array}$$

After applying the ADMM method [40], the above optimization problem can be solved through multiple iterations, which optimize the two objective parts separately. The detailed optimization process is shown in Algorithm 1 and Figure 1b.
**Algorithm 1 The extended RDA model**
1: **Input:** Observed genotype matrix $X\in R^{n\times m}$; Index set of observed entries Ω 2: Initialize $L_{D}\in R^{n\times m}$, $S\in R^{n\times m}$ to be zero matrices, LS←X, and an autoencoder $D\left(E\left(\cdot \right)\right)$ with randomly initialized parameters.
3: **while** True **do**:
4:   $L_{D}=X-S$
5:   $\theta =\underset{\theta }{argmin}\|L_{D}-D_{\theta }{\left(E_{\theta }\left(L_{D}\right)\right)\|}_{2}$
6:   $L_{D}=D_{\theta }\left(E_{\theta }\left(L_{D}\right)\right)$
7:   $S=X-L_{D}$
8:   $P_{\Omega }\left(S\right)=prox\left(P_{\Omega }\left(S\right)\right)$
9:   $c_{1}=\|X-L_{D}-{S\|}_{2}/{\left\|X\right\|}_{2}$ # check the convergence condition
10:  $c_{2}=\|LS-L_{D}-{S\|}_{2}/{\left\|X\right\|}_{2}$ # check the convergence condition
11:  **if**
$c_{1}<\varepsilon$
**or**
$c_{2}<\varepsilon$: **break**
12:  $LS=L_{D}+S$ # update LS for convergence checking in the next iteration
13: **return**
$L_{D}$ and S

#### 2.1.3. Identification of Subclones by Louvain-Jaccard Clustering

The Louvain–Jaccard clustering method [28,29], a hierarchical clustering algorithm, is used to detect communities from large networks and has been widely used for the clustering analysis of single-cell data [25,43]. The specific workflow for the Louvain–Jaccard method is as follows: constructing a k-nearest neighbor network for the cells based on their similarity by the Euclidean distance of their genotype sites; and then clustering cells into different subclones. The Louvain–Jaccard method is conducted very rapidly, which is scalable to large datasets. In addition, the Louvain–Jaccard method has been demonstrated to be robust to the choice of cluster K [25]; therefore, we do not need to specify the number of subclones in advance.

#### 2.1.4. Construct Subclone Evolutionary Tree by Minimum Spanning Tree Method

Following the RDAClone framework [25], we adapted the following two steps to construct a subclone evolutionary tree: (1) the cells belonging to the same subclone should be homogeneous; hence, their genotypes should be almost identical. Specifically, for scSNV data, the genotype with the highest frequency among these cells was regarded as the consensus one, which was treated as subclone genotype; and (2) the Euclidean distance between any pair of subclones based on their subclone genotypes was calculated to find the minimum spanning tree.

### 2.2. Datasets and Preprocessing

**Simulated datasets**: we simulated five groups of data, with each containing five datasets, using the function “simulateData2” in the oncoNEM [21] R package. This is a two-step generation process that consists of constructing a clonal tree structure and simulating genotype observations based on the simulated subclone tree. Specifically, the data of 3000 cells for each of the five groups were simulated by changing one of the following five parameters while keeping the other four parameters fixed: the number of genotype sites (#GS), false-positive rate (FPR), false-negative rate (FNR), missing rate (MR), and the number of simulated subclones (#SC). The detailed parameters for each dataset are shown in Table 1.

For run-time comparison analysis, we simulated 10 datasets by changing the number of cells ranging from 500 to 5000 by 500, the number of genotypes ranging from 200 to 2000 by 200, and the number of subclones ranging from 10 to 100 by 10, with the following fixed parameters: 15% FPR, 15% FNR, and 30% MR.

**Real Data**: Two real scSNV datasets were used in this study: a high-grade serous ovarian cancer (HGSOC) dataset [22,44] and an essential thrombocythemia (ET) dataset from a sample of JAK2-negative myeloproliferative neoplasm [18]. To check whether our model was robust with the high missing rate data, we randomly changed non-missing entries of both datasets to missing, with the proportion ranging from 20.7% to 60.7% by 10% for the HGSOC dataset and 60.7% to 72.7% by 3% for the ET dataset. In this way, five evaluation datasets were generated for each real dataset.

### 2.3. Evaluation Metrics

To compare RDA with other SCGS data analysis methods, RobustClone, BEAM, and SCG, we defined the following four metrics to evaluate the accuracy of recovered genotype data and clustering under the simulated datasets. Specifically, (1) the sum of the false positive rate and false negative rate (FPNR) of the recovered matrix obtained by each method divided by the FPNR of the input matrix was calculated by formula (6); (2) the proportion of missing entries correctly imputed by each method was calculated by formula (7); (3) the proportion of incorrectly recovered genotype matrix entries by each method was calculated by formula (8); and (4) the clustering accuracy based on the ARI [45] by comparing the predicted clustering result based on the recovered matrix and the subclone ground truth was obtained. Formulas (6) through (8) are defined as follows:

(6) $$FPNR=FPR+FNR=\frac{FP}{FP+TN}+\frac{FN}{FN+TP}$$

(7) $$accuracy_{\bar{\Omega }}=\frac{\|P_{\bar{\Omega }}{\left(I\left(L_{D}=X_{true}\right)\right)\|}_{0}}{m\times n\times MR}$$

(8) $$error=\frac{\|I{\left(L_{D}\neq X_{true}\right)\|}_{0}}{m\times n}$$

where FPR and FNR denote the false positive rate and false negative rate, respectively; FP, FN, TP, and TN represent false positive, false negative, true positive and true negative, respectively; I is an indicator function, where when the condition is true I returns 1, and is otherwise 0; $L_{D}$ is the recovered genotype matrix; $X_{true}$ is the corresponding simulated ground truth; MR is the abbreviation of the missing rate; and m and n denote the number of genotype sites and cells, respectively.

In addition, we defined the following evaluation metrics to determine whether these SCGS models could handle real SCGS data with a high missing rate using the 10 generated datasets based on real data. Specifically, (1) the proportion of the additional missing entries correctly recovered by each method was calculated by formula (7); and (2) the ARI between the predicted clustering result based on the genotype matrix recovered by each method and the clustering result from the previous publication were used.

## 3. Results

### 3.1. Model Evaluation and Comparison on the Simulated Datasets

**Figure 2:**
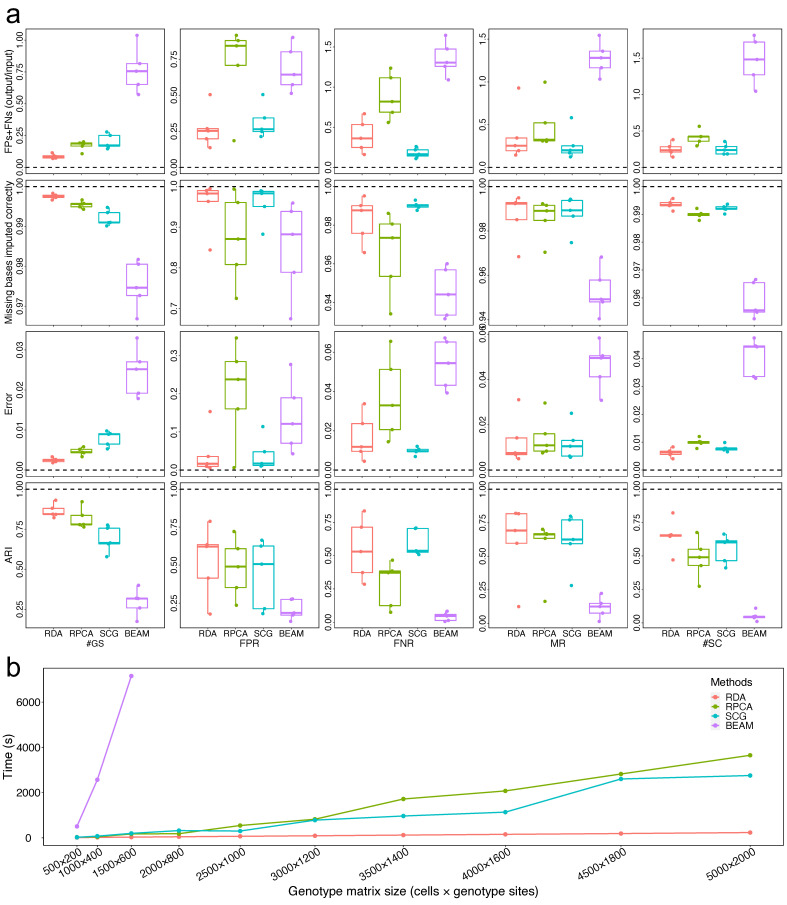
Genotype matrix recovery accuracy, clustering, and run-time comparison on the simulated datasets. (**a**) The matrix recovery accuracy (evaluated by FPs + FNs, missing bases imputed correctly, error), and clustering accuracy (evaluated by ARI between the known clustering and the predicted clustering based on recovered matrix) for each method. (**b**) Run-time comparison for fitting four models on the ten simulated datasets with different sizes of cells, genotypes and subclones. Clearly, the computational complexity of RDAClone is almost linear against the matrix size, which is much efficient comparing with other methods.

We compared RDAClone with several state-of-the-art methods, including RobustClone, SCG (doublet-naïve variant), and BEAM, for the recovery of the genotype matrix and its clustering based on the defined four metrics using the simulated datasets. Because BEAM has difficulty handling large-scale datasets, we subsampled the input matrix (5 repeats) for the BEAM method. As summarized in Figure 2a, (1) for the five simulated datasets with different #GS, the accuracy of the genotype matrix recovered by RDAClone was higher than the other three methods, and the clustering accuracy of RDAClone were higher than those of the other three methods. (2) RDAClone had a comparable performance to SCG for matrix recovery and clustering, and these two methods were better than RobustClone and BEAM under the five simulated datasets with varying FPRs. (3) RDAClone performed better than RobustClone and BEAM under the simulated datasets with changing FNR and was slightly weaker than SCG. Finally, (4) with the varying of MR or the number of subclones, the accuracy of matrix recovery and clustering for RDAClone was slightly higher than that of SCG and these two methods performed better than RobustClone and BEAM for both matrix recovery and clustering (Figure 2a). Additionally, we also evaluated the genotype matrix recovery accuracy using precision, recall and F1 score (Figure S1) and the clustering accuracy using normalized mutual information (NMI) and V measure (Figure S2). These results further supported the superiority and robustness of RDAClone. Furthermore, we performed sensitivity analysis on the resolution of Louvain-Jaccard clustering controlling the number of resulting clusters and found the clustering accuracy was less likely to be affected by resolution (Figure S3).

In addition, to assess the scalability of these methods, we simulated 10 datasets with different numbers of cells, genotypes, and subclones. Generally, RDAClone, RobustClone, and SCG could be scaled to large datasets, but RDAClone performed the fastest across all simulated datasets (Figure 2b) owing to its reliance on a fixed number of cells at each iteration of iterative stochastic optimization (Figure 2b). The computational complexity of RDAClone was almost linear against the matrix size, which was much more efficient compared with the other methods.

### 3.2. RDAClone Accurately Recovers Missing Entries and Identifies Subclones on a Real scSNV Dataset

**Figure 3:**
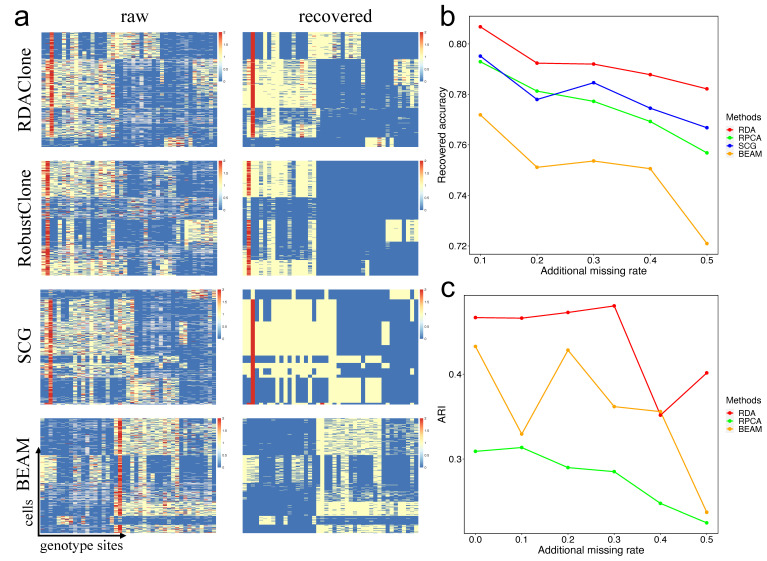
Genotype matrix recovery, and cell clustering on the HGSOC dataset. (**a**) The heatmap of raw and recovered genotypes matrix for each of methods: RDAClone, RobustClone, SCG and BEAM. The ordering of cells and genotype sites based on the clustering of recovered matrix. (**b**) The proportion of missing entries correctly recovered by RDAClone, RobustClone, SCG and BEAM under five sparsity data generated by additional missing rate. (**c**) The clustering accuracy was evaluated by ARI between the published SCG results and clustering results of recovered matrix by RDAClone, RobustClone and BEAM.

We next evaluated the extent to which the genotype matrix recovered by the extended RDA could identify real subclones. We performed RDAClone on the HGSOC dataset (a real scSNV dataset) consisting of 420 cells × 43 genotype sites with a 10.7% missing rate, with the other three methods, RobustClone, SCG, and BEAM, as a comparison [22,44]. The results showed that RDAClone could cluster 420 cells into five subclones, which contained 111, 99, 93, 82, and 35 cells, and 43 genotype sites into three blocks, which contained 18, 18, and seven genotype sites. The heatmaps of the recovered matrix and raw matrix for each method are shown in Figure 3a, and the ordering of cells and genotype sites is based on the predicted clustering result.

To test whether RDAClone was robust with sparse data, five datasets were generated by randomly converting non-missing entries of the HGSOC dataset to missing so that the missing data ranged from 20.7% to 60.7% in increments of 10%. By comparison, we found that RDAClone could recover missing entries with the highest accuracy and was robust relative to the other three methods under data with various levels of sparsity (Figure 3b). Due to the lack of the real subclone structure for the HGSOC dataset as a benchmark, we used the published clustering result of the SCG model as the ground truth for the clustering assessment. ARI was applied to compare the clustering accuracy between RDAClone, RobustClone, and BEAM. We found that the clustering accuracy of RDAClone was significantly higher than RobustClone and BEAM, and RobustClone performed the worst (Figure 3c).

### 3.3. RDAClone Works Well on a Real scSNV Dataset with a High Missing Rate 

**Figure 4:**
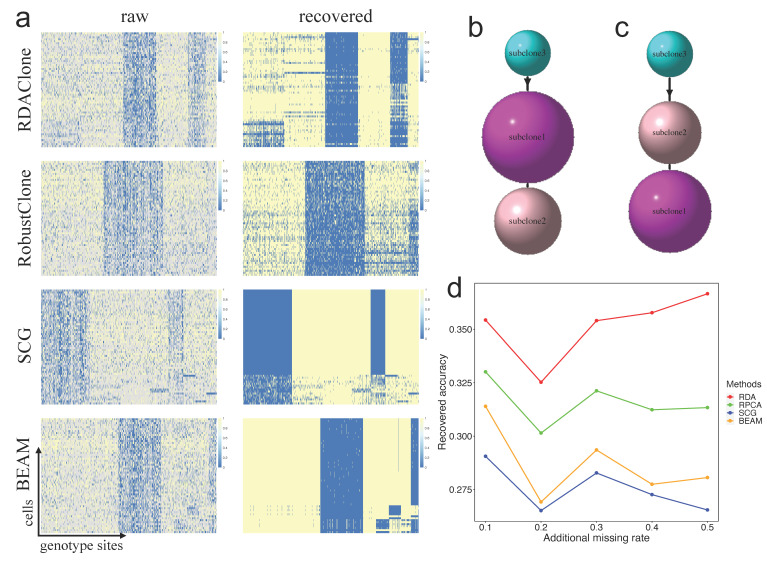
RDAClone works well on ET dataset with a high missing rate. (**a**) The heatmap of raw and recovered genotype matrix for each of methods: RDAClone, RobustClone, SCG and BEAM. The ordering of cells and genotype sites based on the clustering of recovered matrix. (**b**) The evolutionary tree inferred from the recovered matrix by RDAClone. (**c**) The evolutionary tree inferred from the recovered matrix by RobustClone. (**d**) The proportion of missing entries correctly recovered by RDAClone, RobustClone, SCG and BEAM under five sparsity data generated by additional missing rate.

We next demonstrated how RDAClone could recover a high missing rate genotype matrix to infer real subclone relationships. We applied RDAClone on the ET dataset (a real scSNV dataset), which consisted of 58 cells × 712 genotype sites with a 57.7% missing rate, with the other three methods, RobustClone, SCG, and BEAM, used as a comparison [18]. The results showed that RDAClone could cluster 58 cells into three subclones, which contained 26, 19, and 13 cells, and 712 genotype sites into six blocks, which contained 168, 166, 133, 130, 71, and 44 genotype sites. The heatmaps of the recovered matrix and raw matrix for each method are shown in Figure 4a, and the ordering of cells and genotype sites are based on the predicted clustering results. In addition, the linear evolutionary relationship among three subclones could be briefly described as follows: subclone 3 was considered as a normal genotype (root of the evolutionary tree), and the mutation of some genotype sites converted some cells from subclone 3 to subclone 1, and then to subclone 2, as shown in Figure 4b. The RobustClone model had a similar linear evolutionary relationship between subclones to RDAClone, but some cells from subclones 1 and 2 were clustered into different subclones (Figure 4c).

We further evaluated the extent to which RDAClone could process the scSNV data with a higher missing rate. We randomly dropped out the non-missing entries of the ET dataset to missing so that the missing rate ranged from 60.7% to 72.7% with the increments of 3%, which generated five datasets. The results showed that RDAClone recovered missing entries with the highest accuracy and was robust relative to the other three methods under data with various levels of sparsity (Figure 4d).

## 4. Discussions

SCGS data provides unprecedented insights to characterize intra-tumor heterogeneity and infer tumor subclone evolutionary relationships. However, the sequencing errors resulting in FP, FN, and MB severely limit its application. In this study, we proposed a deep learning framework, RDAClone, for SCGS data analysis. RDAClone can be utilized to recover a genotype matrix from corrupted observations even with a high missing rate, cluster cells into subclones based on the recovered matrix, and then perform subclone relationship inference to construct a subclone evolutionary tree.

RDAClone is a nonlinear version of RobustClone, which has been shown to be a powerful and efficient tool for SCGS data analysis compared to most previous methods [22,24,25]. Based on our comparison, we found that the matrix recovery accuracy of RDAClone was significantly higher than that of RobustClone on the simulated and real datasets, as well as the subsequent clustering performance based on the recovered genotype matrix. In addition, RDAClone can be scaled to super large datasets of $10^{7}$ entries with a run time of no more than 231 s, but RobustClone has a run time of up to 3649 s because RDAClone relies on a fixed number of cells at each iteration of iterative stochastic optimization. RDAClone also works well on high-missing rate scSNV data, i.e., with higher accuracy.

RDAClone has three components, i.e., extended RDA, the clustering method, and the evolution inference method. Each of these components can be adapted to other applications. Recent studies emerged to infer subclone evolution from single-cell RNA sequencing (scRNA-seq) data due to its high input amount [46,47,48,49]. The sparsity and noise are general for single-cell data (e.g., scRNA-seq and single-cell chromatin accessibility data) and, thus, our extended RDA can be also applied to other areas of single-cell data analysis. In addition, by combining with the cell specific network method [50], RDAClone can be adapted to analyze the cellular and gene networks for potential applications [51,52,53,54,55] in biology and medicine.

## Figures and Tables

**Table 1:** Simulation parameters for datasets of five groups.

	#CELL	#GS	FPR	FNR	MR	#SC
Group 1	3000	From 1200 to 2000 by 200	0.15	0.15	0.3	50
Group 2	3000	1000	From 0.2 to 0.4 by 0.05	0.15	0.3	50
Group 3	3000	1000	0.15	From 0.2 to 0.4 by 0.05	0.3	50
Group 4	3000	1000	0.15	0.15	From 0.4 to 0.8 by 0.1	50
Group 5	3000	1000	0.15	0.15	0.3	From 60 to 100 by 10

## Data Availability

The data presented in this study are available on request from the corresponding author.

## Supplementary Materials

The following are available online at https://www.mdpi.com/article/10.3390/genes12121847/s1. Figure S1: Genotype matrix recovery accuracy comparison on the simulated datasets, Figure S2: Clustering accuracy comparison on the simulated datasets, Figure S3: Sensitivity analysis on the resolution (default to 1.0) of Louvain-Jaccard clustering.
