# Supplementary material for: RDAClone: Deciphering Tumor Heterozygosity through Single-Cell Genomics Data Analysis with Robust Deep Autoencoder
Source: Genes (Basel). 2021 Nov 23;12(12):1847. doi: 10.3390/genes12121847 (PMC8701080; doi:10.3390/genes12121847)
Supplement: Supplementary file 1 [file genes-12-01847-s001.zip › supplementary/SI.pdf]

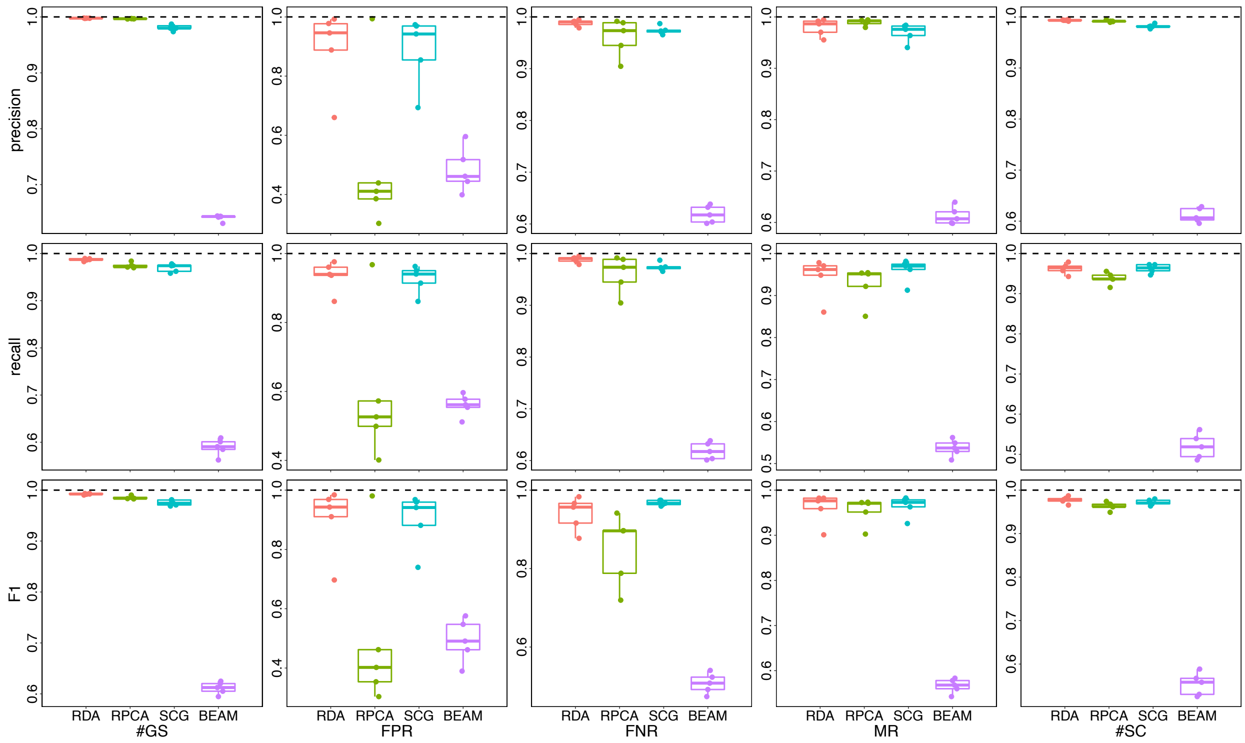

Figure S1. Genotype matrix recovery accuracy comparison on the simulated datasets. The matrix recovery accuracy was evaluated by precision, recall, F1 score.

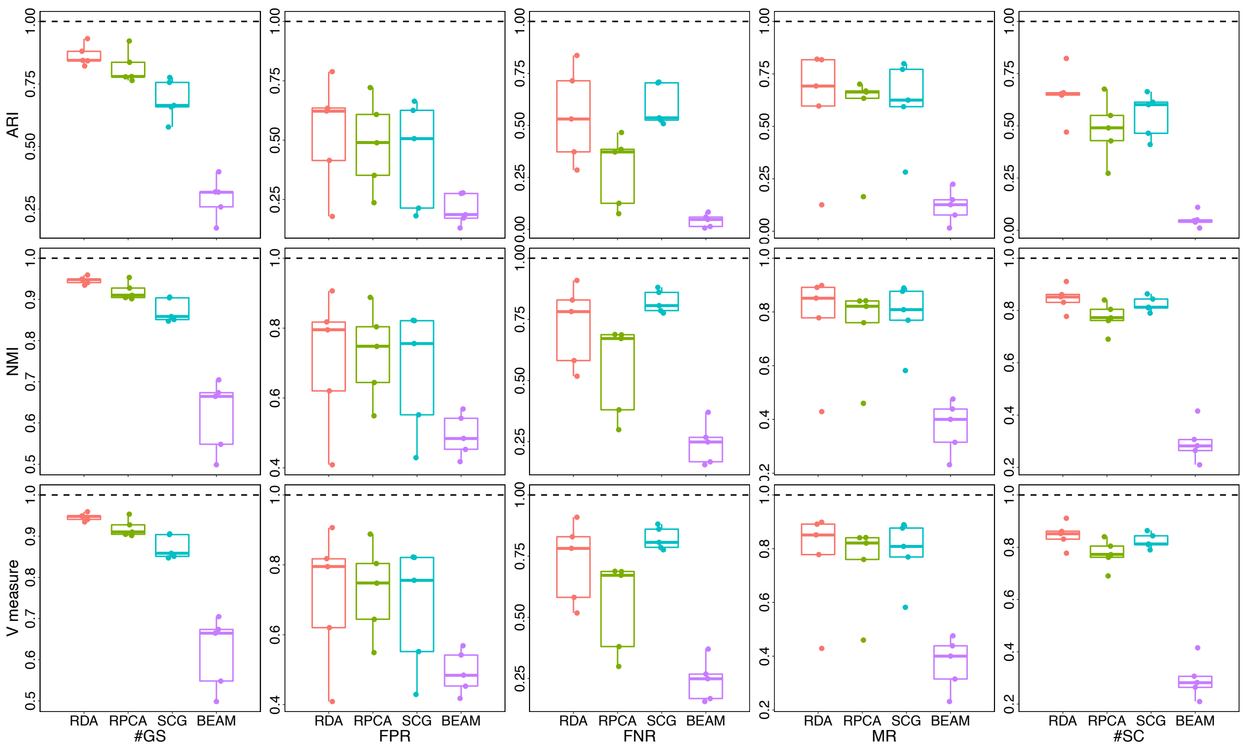

Figure S2. Clustering accuracy comparison on the simulated datasets. The clustering accuracy was evaluated by ARI, NMI and V measure between known clustering and predicted clustering based on recovered matrix.

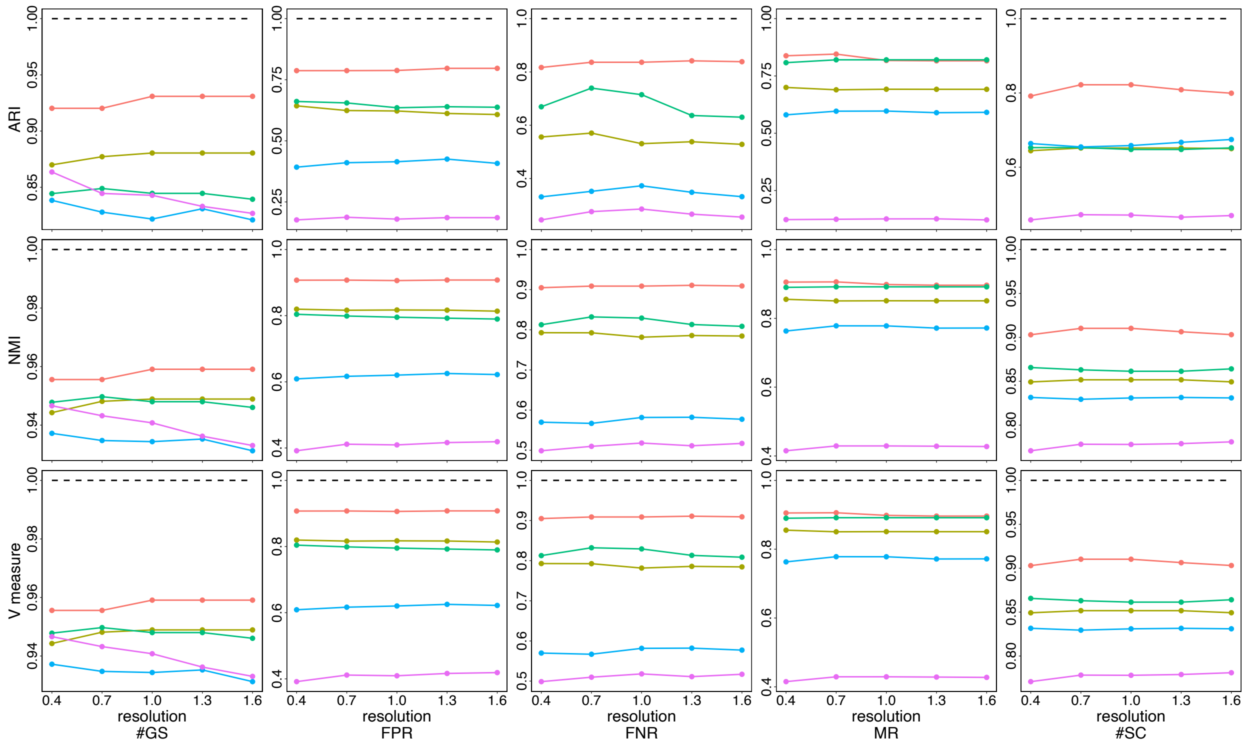

Figure S3. Sensitivity analysis on the resolution (default to 1.0) of Louvain-Jaccard clustering. The clustering accuracy was evaluated by ARI, NMI and V measure between known clustering and predicted clustering based on recovered matrix across various resolution settings.
